# The Effect of Sn-2 Palmitate on Blood Glucose, Lipids and Body Composition in Middle-Aged and Elderly Adults: A Randomized, Double-Blinded Controlled Trial

**DOI:** 10.3390/nu16070973

**Published:** 2024-03-27

**Authors:** Wuxian Zhong, Ai Zhao, Xuetao Wei, Shuai Mao, Pin Li, Qianqian Shen, Hong Zhang, Hua Jiang, Peiyu Wang, Yumei Zhang

**Affiliations:** 1 Department of Nutrition and Food Hygiene, School of Public Health, Peking University, Beijing 100191, China; zhongwuxian@pku.edu.cn (W.Z.); maoshuai@pku.edu.cn (S.M.); 2111110222@pku.edu.cn (P.L.); qshen@stu.pku.edu.cn (Q.S.); 2 Vanke School of Public Health, Tsinghua University, Beijing 100084, China; 3 Department of Toxicology, School of Public Health, Peking University, Beijing 100191, China; weixt@263.net; 4 Wilmar (Shanghai) Biotechnology Research & Development Center Co., Ltd., Shanghai 200137, China; zhanghongsh@cn.wilmar-intl.com; 5 School of Nursing, Peking University, Beijing 100191, China; 6 Department of Social Medicine and Health Education, School of Public Health, Peking University, Beijing 100191, China

**Keywords:** sn-2 palmitate, palmitic acid, glucose, high-density lipoprotein cholesterol, low-density lipoprotein cholesterol, body composition, skeletal muscle mass index

## Abstract

Sn-2 palmitate is widely used in infant formula. However, little is known about its effects on metabolism and body composition in middle-aged and elderly adults. In a double-blinded, randomized controlled trial, we enrolled Chinese adults aged 45–75 years with self-reported constipation. Individuals were randomly assigned in a 1:1 ratio to a 1,3-dioleoyl-2-palmitoyl-glycerol (OPO)-enriched oil (66% palmitic acid in the sn-2 position) or a control vegetable oil (24% palmitic acid in the sn-2 position) daily for 24 weeks. Skim milk powder was used as the carrier for both fats. Interviews and body composition were performed at baseline, week 4, week 12 and week 24. A fasting blood draw was taken except at week 4. This study was a secondary analysis and considered exploratory. A total of 111 adults (83 women and 28 men, mean age 64.2 ± 7.0 years) were enrolled, of whom 53 were assigned to the OPO group and 57 to the control group. During the intervention, blood glucose, triglyceride, the triglyceride-glucose index, total cholesterol, low-density lipoprotein cholesterol and remnant cholesterol remained stable, while high-density lipoprotein cholesterol decreased in both groups (*p* = 0.003). No differences in change were observed between the groups (all *p* > 0.05). From baseline to week 24, the level of visceral fat increased slightly (*p* = 0.017), while body weight, total body water, protein, soft lean mass, fat-free mass, skeletal muscle and skeletal muscle mass index (SMI) decreased in two groups (*p* < 0.01). At weeks 4, 12 and 24, the SMI decreased less in the OPO group than in the control group, with a trend towards significance (*p* = 0.090). A 24-week daily intake of sn-2-palmitate-enriched oil had no adverse impact on fasting blood glucose, lipids and body composition compared with the control vegetable oil in Chinese adults (funded by Chinese Nutrition Society National Nutrition Science Research Grant, National Key Research and Development Program of China and Wilmar (Shanghai) Biotechnology Research & Development Center Co., Ltd.; ChiCTR1900026480).

## 1. Introduction

Sn-2 palmitate is a modified or synthetic triacylglycerol (TAG) with palmitic acid (PA) esterified mainly (about 75%) at the sn-2 position [1]. The 1,3-dioleoyl-2-palmitoylglycerol (OPO), a major sn-2 palmitate, has been widely used in infant formulas as a human milk fat substitute [1]. Upon hydrolysis, OPO forms sn-2 monoglyceride, which is water soluble and easily absorbed. A recent meta-analysis demonstrated that compared with standard formula, Sn-2-palmitate-enriched formula feeding could effectively promote weight gains, bone mineral accumulation and stool fatty acid (FA) soap reduction in infants [2]. The generation of saturated FA soaps has been proven to be a major cause of hard stools [3]. Additionally, an animal study found that sn-2 palmitate was beneficial for the host microbial ecosystem [4]. As osteoporosis [5] and constipation [6] affect middle-aged and elderly people as well, the consumption of OPO may have a similar health-promoting effect on them.

As a kind of TAG, OPO might affect metabolism while exerting a nutritional role, which has been called the “Sn-2 Hypothesis” [7]: saturated FAs located at the sn-2 position of dietary TAGs are more likely to raise the LDL concentration than the same FAs located at the sn-1 or sn-3 positions. However, several studies showed different results. A 6-week cross-over study found that sn-2 palmitate had little effect on lipoprotein concentrations compared with the control [8]. Another randomized study showed that fats with a higher proportion of PA at the sn-2 position decrease postprandial lipemia in healthy subjects [9]. Studies by Filippou et al. [10,11] suggest that sn-2 palmitate did not have adverse effects on insulin secretion and glucose homeostasis or the insulin and glucose response to meals in healthy adults. In these studies, which are focused on a young population [8,9,10,11], high doses (28 or 50 g) of test fat were consumed, which is higher than the daily intake. Additionally, there was limited knowledge regarding the metabolic effects of long-term and low-dose intake in middle-aged and elderly individuals when considering sn-2 palmitate as a potential food option.

Apart from traditional biomarkers such as blood glucose, total cholesterol (TC), high-density lipoprotein cholesterol (HDLC) and low-density lipoprotein cholesterol (LDLC), current studies have confirmed that the triglyceride-glucose (TyG) index can be used as a reliable and convenient surrogate for insulin resistance, and it has shown mild to moderate predictive value for cardiovascular disease (CVD) [12]. In addition, studies have also shown that remnant cholesterol (RC) [13] and TC/HDLC ratio [14] are associated with CVD and atherosclerosis. Additionally, body composition, measured by bioelectrical impedance analysis (BIA) [15], can provide information beyond body mass index (BMI) [16]. For example, the appendicular skeletal muscle mass can be evaluated and used to calculate the skeletal muscle mass index (SMI) and for the diagnosis of sarcopenia in older adults [17]. However, limited research has been conducted on the impact of sn-2 palmitate on body composition.

Therefore, the purpose of this study was to investigate the impact of consuming sn-2 palmitate daily compared with control fat on fasting glucose, lipids, and body composition in a randomized controlled trial (RCT). We hypothesized that sn-2 palmitate would mildly increase lipoprotein concentrations and body fat mass in middle-aged and elderly adults.

## 2. Materials and Methods

### 2.1. Participants

This study was based on a single-center, parallel-arm, double-blinded, 24-week RCT (registered at www.chictr.org.cn, ChiCTR1900026480), which was conducted at Peking University Health Science Center (PKUHSC) from December 2019 to September 2023, and formal screening began from February 2022 due to COVID-19. This RCT aimed to compare the dietary intake of OPO (66% PA in the sn-2 position) compared with a control fat (24% PA in the sn-2 position) on constipation improvement and areal bone mineral density at lumbar spine, total hip, or femoral neck in adults aged 45–75 years. The secondary outcomes of the trial included blood glucose, lipids and body composition. Participants were recruited from several communities in Beijing. The inclusion criteria are as follows: (1) male or female aged 45–75 years; (2) self-reported constipation (which can be manifested as a decrease in the frequency of spontaneous bowel movements, straining during defecations, lumpy or hard stools, sensation of incomplete evacuation or anorectal obstruction/blockage, and manual maneuvers to facilitate defecations); (3) not underweight or severely obese [18.5 ≤ BMI (in kg/m^2^) < 40]; (4) no abnormal liver function or renal function; (5) willing to intake milk powder daily, and cooperate in questionnaire completion and biological sample collection. Individuals were not in the acute phase of chronic diseases and without severe metabolic diseases (for example, complications of diabetes) or chronic intestinal inflammation. Participants who have had gastrointestinal or rectal surgery or have a history of gastrointestinal malignancy were excluded. In particular, participants must not be allergic to milk powder or lactose intolerant, and during the intervention, there must not be dramatic changes in diet and lifestyle, like starting a weight loss plan.

### 2.2. Intervention

**Figure 1:**
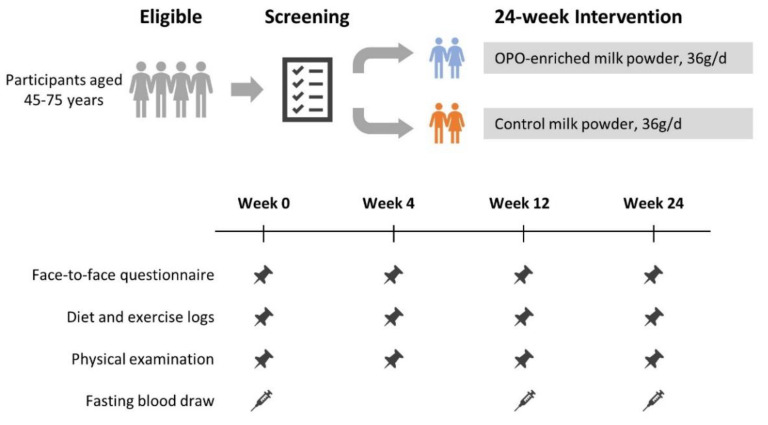
The study procedure. The symbols mean different items investigated at different timepoints, pushpins are for questionnaire survey and physical examination, and syringes are for blood draw.

The OPO-enriched oil and a mixture of vegetable oils were used as intervention and control oil, separately. The detailed fatty acid composition of these two fats is shown in Table 1. The two oils had similar contents of palmitic acid, oleic acid and linoleic acid, with different proportions of sn-2 palmitate. Considering the acceptability of intervention over 24 weeks, skim milk powder was used as a carrier. Both intervention and control milk powders were made up of a mixture of 75% skim milk powder and 25% fat, with each 100 g of milk powder containing 40.9 g of carbohydrates, 24.7 g of protein and 25.6 g of fat, providing 495.5 kcal of energy. Two packets (36 g) of milk powder per day, equivalent to approximately 300 g of liquid milk, were consumed, and participants were asked to keep the packages until the next visit. Detailed study procedures are outlined in Figure 1. Face-to-face questionnaires and physical examination were performed at week 0 (baseline), week 4 (follow-up 1), week 12 (follow-up 2) and week 24 (follow-up 3), and a fasting blood draw was taken except at week 4 to assess metabolic biomarkers.

### 2.3. Screening and Eligibility

Recruitment occurred through online advertisements by WeChat and offline fliers distributed in the community near PKUHSC in Beijing. Preliminary screening was completed via phone or face-to-face inquiry. If eligibility criteria were met, individuals were invited to complete a physical exam and evaluation of medical health history, and results of liver and kidney function from the fasting blood draw were reviewed to determine eligibility. The study was approved by the institutional review boards at Peking University (IRB00001052-19097), and participants signed an approved informed consent.

### 2.4. Randomization and Blinding

The random number list was generated using “PROC PLAN” in SAS by an independent study staff person who was not involved in enrollment or outcome assessment. Sealed opaque envelopes were used for allocation concealment. Randomization was stratified by age (<60 years vs. ≥60 years) and gender. Within each stratum, balanced block randomization was used with a block size of 4 and a treatment allocation ratio of 1:1 to an sn-2 palmitate milk powder and a control milk powder. The properties and packaging of these two milk powders were identical, distinguished by the letters A and B on the label. They were provided by Wilmar (Shanghai) Biotechnology Research & Development Center, and the correspondence information between the letter and the milk powder was also determined and kept by them. During the intervention, both participants and investigators were masked to the treatment received.

### 2.5. Anthropometric Assessment

Height was measured to the nearest 0.1 cm using a stadiometer with a fixed vertical backboard and an adjustable head piece (Seca, Hamburg, Germany). Total body weight was measured to the nearest 0.1 kg on a bioimpedance analysis instrument (InBody 370, Biospace, Seoul, Republic of Korea). Body mass index (BMI) was calculated as the body mass divided by height squared, and body composition was measured by BIA to assess the total body water, protein, soft lean mass, fat-free mass, skeletal muscle, body fat mass, InBody score (a composite health assessment score by InBody) and visceral fat level. Skeletal muscle mass index (SMI) was computed as the appendicular skeletal muscle mass divided by height squared [17]. Systolic and diastolic blood pressure were measured to the nearest 1 mmHg using a portable electronic blood pressure monitor (HEM-7124, OMRON, Kyoto, Japan).

### 2.6. Blood Serum Assessment

At baseline, midterm and endpoint, blood samples were collected between 8 a.m. and 10 a.m. after at least 10 h of fasting, then samples were transferred to our laboratory as soon as possible and centrifuged for 10 min at 3000× *g* rpm. The serum was stored at −80 °C until measurements of blood glucose (GLU), triglyceride (TG), TC, HDLC and LDLC were taken. These biomarkers were performed on an automatic biochemistry analyzer (Hitachi 7180, Tokyo, Japan): GLU was determined by the hexokinase method, TC and TG concentrations were assessed by using enzymatic procedures (CHOD-PAP and GPO-PAP, respectively), and HDLC and LDLC were determined by the direct clearance method (catalase). The TyG index was calculated as Ln [fasting TG (mg/dL) × fasting glucose (mg/dL)/2] [12]. RC measures were computed as TC minus HDLC and LDLC [18].

### 2.7. Medical History and Lifestyle Behavior

Self-reported medical history was investigated by face-to-face questionnaires at baseline; participants were asked whether they had been diagnosed with hypertension, diabetes mellitus or dyslipidemia before the trial. At each time point, lifestyle behavior, including exercise and smoke exposure, was evaluated. Physical activity level over the last week was assessed by using the validated Chinese version of the International Physical Activity Questionnaire—Short Form (IPAQ-SF), and MET-minutes per week were calculated and divided into lower, medium and higher levels [19]. Smoking status (yes or no) and second-hand smoke exposure (less than 1 day/week or at least 2 days/week, defined as exposure to tobacco smoke from someone nearby for >5 min/day) were self-reported.

### 2.8. Diet Assessment

Dietary nutrient and food intake was assessed by a 3 d diet recall (1 weekend day and 2 weekdays) and a semi-quantitative food frequency questionnaire (FFQ), separately. Unified training of interviewers was completed prior to the survey. Participants were instructed to report all food and beverages consumed in detail (standard-sized bowls, teaspoons and illustrated photos of food items were shown in order to improve the estimation accuracy). The data from the 3 d diet logs were coded and calculated for total energy, macronutrients and micronutrients based on the Chinese Food Composition Table [20]. Daily kilocalories and nutrients consumed were averaged. The modified FFQ used in the current study was derived from the questionnaire used in our research group [21], which contains 33 food items, including staple foods, vegetables, fruits, seeds and nuts, soybean, livestock and poultry meat, seafood, eggs, dairy, cooking oil and condiments. Consumption frequency (daily, weekly, monthly or never) and average consumption weight of each food were investigated, and daily dietary intake over the previous month was computed.

At baseline, participants were required to maintain their dietary habits during the intervention period, except for the increased intake of milk powder and the corresponding reduction in the intake of other dairy products, which was brought about by the intervention. Additionally, no extra nutrition education or counseling was provided.

### 2.9. Statistics

This trial was powered to detect a difference in the change of score of constipation symptoms between the groups, providing 80% power to detect a 0.6-point difference at a 0.025 level of significance, assuming a standard deviation of 1.0 and 15% dropouts [22]. In the secondary analysis of blood glucose, lipids and body composition, which were considered exploratory, no such calculations were performed. There was no control for multiple hypothesis testing, and no formal adjustment was made to the *p* values or confidence intervals. Thus, results should be judged with caution.

All analyses were performed on an intention-to-treat (ITT) basis using all randomized patients with at least one follow-up. Mean ± SD and median (interquartile range) were used to summarize demographic and clinical characteristics. Categorical variables were compared between groups using the Chi-square test or the Fisher exact test. Continuous variables were compared between groups using the Student’s *t*-test or Wilcoxon rank sum test. To compare the effects of the intervention on those metabolic indicators mentioned above, we used a general linear mixed-effects model (GLMM) fit to the longitudinal observations at 4 time points during the study with random subject-level intercept and fixed effects of time, study group, and group × time interaction. For variables that were not normally distributed, log transformation was used. All results were analyzed unadjusted and adjusted for age and gender. Since GLMM offers a simple alternative to handling missing data under MAR without imputation, and there were fewer missing data in this study, no data were imputed. Further subgroup analysis (non-prespecified and considered exploratory) was performed according to gender and medical history (dyslipidemia) at baseline. Statistical analyses were conducted using SAS software version 9.4 (SAS Inc., Cary, NC, USA).

## 3. Results

**Figure 2:**
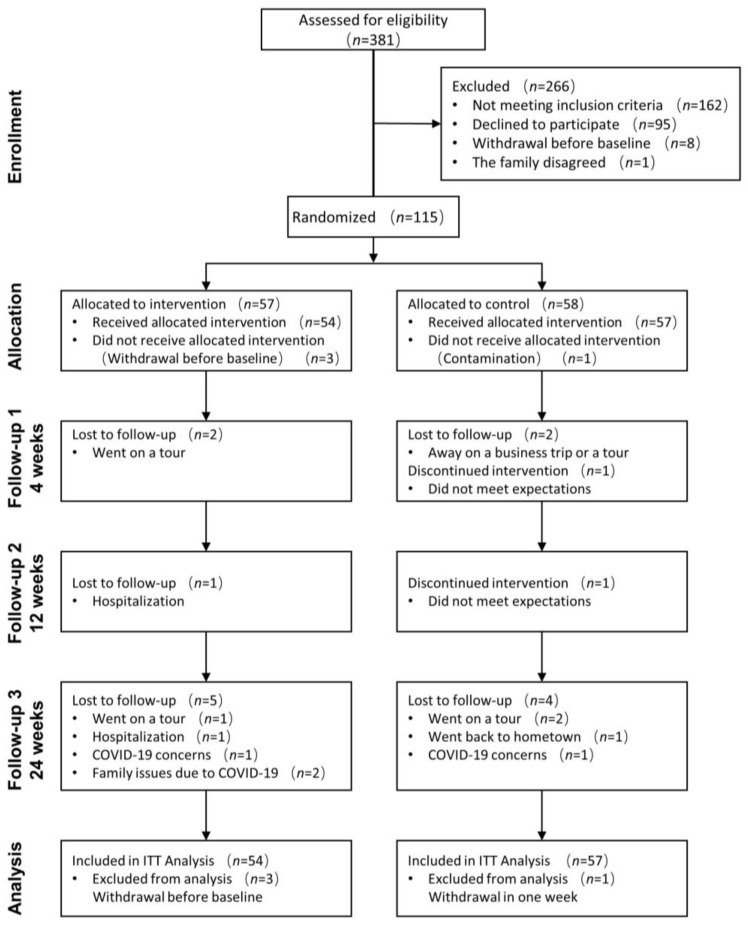
Consolidated Standards of Reporting Trials diagram depicting number of participants enrolled at each study phase and the reasons for dropout. Two participants in the control group dropped out of the study early. At the last follow-up point, 9 participants (5 in the OPO group, 4 in the control group) withdrew due to personal reasons or concerns about COVID-19.

The intervention and follow-up were conducted from March 2022 to September 2023. After initial screening, 30 men and 85 women were randomly allocated into one of two groups: (1) OPO group (*n* = 57) or (2) control group (*n* = 58) (Figure 2). Three individuals quit before the baseline interview, and another person in the control group quit after one week. Finally, 111 participants (54 in OPO and 57 in control) were included in the ITT analysis. Compliance, judging by the standard of no less than 80% consumption of milk powder, was 90.7% in the OPO group and 91.2% in the control group.

Demographic characteristics were balanced among the two groups, as shown in Table 2. The average age of the participants was 64.2 ± 7.0 years old (range: 47–75 y). The study population consisted of 83 women, most of whom were postmenopausal. The average BMI of the participants was 25.0 ± 3.3 kg/m^2^, and 57.4% of participants were overweight or obese. Most of the population were well educated and non-smokers. The exposure rate of second-hand smoke was very low. Additionally, 30.6% and 26.1% of individuals reported a medical history of dyslipidemia and hypertension, and 8.1% of those reported diabetes mellitus. The intake of energy, fat, protein, micronutrients and food groups at baseline were balanced between the two groups, except for the slightly higher intake of carbohydrates and yogurt in the OPO group (Table 3, Supplemental Table S1).

**Figure 3:**
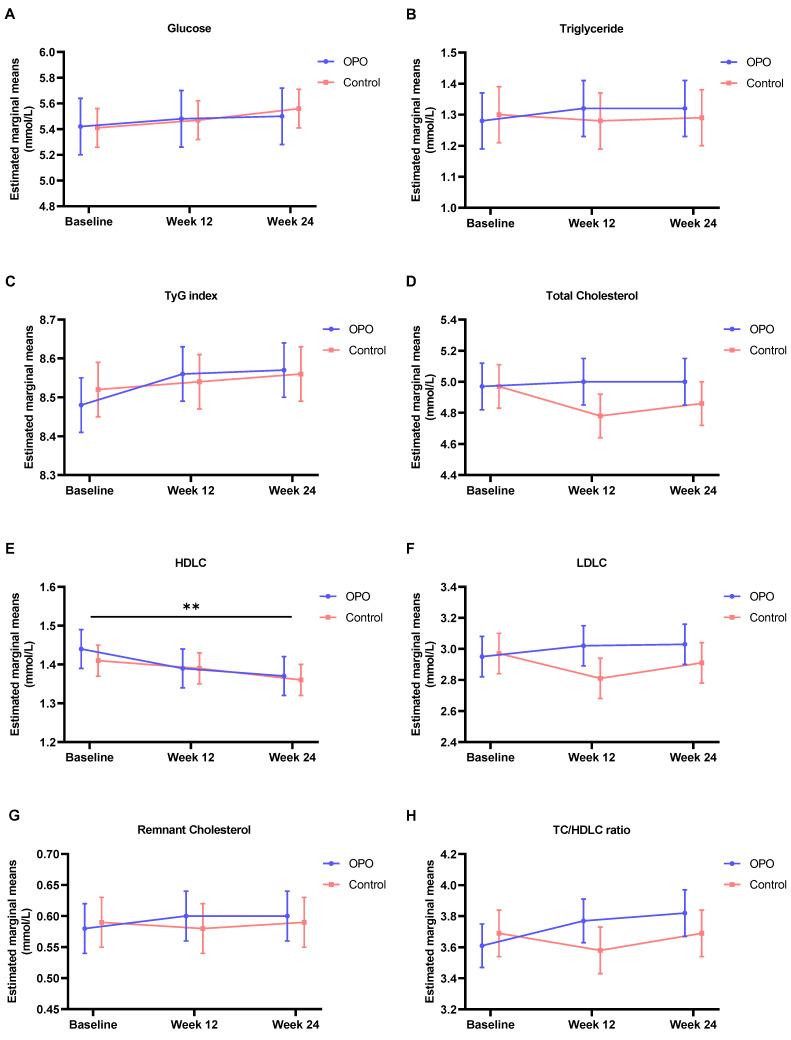
The trajectories of adjusted mean changes in blood glucose and lipids (**A**–**H**) during the study in two arms. TyG index, triglyceride-glucose index; TC, total cholesterol; HDLC, high-density lipoprotein cholesterol; LDLC, low-density lipoprotein cholesterol. ** *p* < 0.01.

Blood glucose and lipids were balanced in two groups at baseline (*p* > 0.05). Over 24 weeks, blood glucose, triglyceride, the TyG index, total cholesterol, LDLC, remnant cholesterol and TC/HDLC ratio remained stable in both groups (main effect of time, all *p* values > 0.05; Figure 3 and Table 4). However, HDLC decreased during the intervention in both the OPO and control groups (main effect of time, *p* = 0.003). At week 24, participants in the OPO group had a −0.07 (SE 0.02) mmol/L decrease in HDLC compared with those in the control group, who had a −0.05 (SE 0.03) mmol/L decrease (Supplemental Table S2). At week 12, participants in the OPO group had a greater TH/HDLC ratio increase than those receiving the control: adjusted mean difference (95% confidence interval, CI) 0.22 (0.00, 0.45), *p* = 0.049. But, at week 24, the mean difference was not significant. Further adjustment for baseline carbohydrates and yogurt consumption in the GLMM yielded the same results.

At baseline, all body composition indicators were balanced in the OPO and control groups (all *p* values > 0.05). Over the course of the intervention, body weight, BMI, total body water, protein, soft lean mass, fat-free mass, skeletal muscle and SMI decreased (main effect of time, all *p* values < 0.01; Table 5 and Supplemental Figure S1) in both arms, whereas visceral fat level increased slightly (main effect of time, *p* = 0.017) and body fat mass, percent body fat and InBody score remained stable. A group × time interaction tended to be significant for SMI (*p* = 0.090), and at weeks 4, 12 and 24, the SMI rose in the OPO group compared with the control group: adjusted mean differences (95% CI) were 0.05 (−0.15, 0.25), 0.16 (−0.04, 0.36) and 0.17 (−0.04, 0.37), respectively, and all *p* values > 0.05 (Supplemental Table S3). Further adjustment for baseline carbohydrates and yogurt consumption in the GLMM obtained the same results.

Subgroup analyses showed that there was the same trend for HDLC (*p* = 0.001, Supplemental Tables S4 and S5) when comparing the control with the OPO group in female but not in male participants. In participants with or without dyslipidemia, HDLC decreased, and no group × time interaction was observed. Instead, an insignificant trend was found for SMI in those without dyslipidemia: at weeks 12 and 24, participants without dyslipidemia in the OPO group had a greater increase in SMI compared with the control, and adjusted mean differences (95% CI) were 0.24 (−0.01, 0.49) and 0.23 (−0.02, 0.48), respectively (*p* = 0.059 and 0.067, Supplemental Table S5). Other indicators of body composition were generally consistent with the main results (Supplemental Table S6).

Over the course of the trial, the physical activity levels and daily intake of staple foods (cereals, potatoes and whole grains), vegetables and fruits remained stable (Supplemental Table S7), and there was a mild decrease in the intake of nuts, seeds and yogurt. A relatively huge decrease in liquid milk intake and a compensatory increase in milk powder intake were observed, which was consistent with the intervention. No significant interactions between group and time were found.

## 4. Discussion

In light of the widespread use of sn-2 palmitate, the aim of the current study was to investigate the impact of OPO-enriched oil on blood glucose, lipids and body composition compared with control oil. Contrary to our hypothesis, we did not observe an adverse effect of the long-term and low-dose intake of OPO on fasting lipids and body fat mass. Although HDLC decreased mildly after the intervention, the mean difference between the two groups was insignificant at each time point. At weeks 12 and 24, participants in the OPO group without dyslipidemia showed a tendency towards a lesser decrease in SMI compared to the control group.

Our findings indicate that PA in the sn-2 position did not affect glucose levels, which is consistent with previous studies that measured postprandial glucose [10,11,23]. However, the insulinotropic polypeptide response decreased after interesterified palm olein (IPO) compared to palm olein (PO) in both healthy young adults and middle-aged type 2 diabetes patients [11,23]. A previous study by Sanders et al. [9] found that IPO resulted in less postprandial triacylglycerol but higher TG at 8 h compared with PO, but in the current study, we did not find an impact of OPO on fasting TG. Due to the stable trajectories of GLU and TG, over the course of the study, neither a time trend nor a time × group interaction of the TyG index was observed in the two groups, which indicates the safety of sn-2 palmitate for glycemic homeostasis.

According to the “Sn-2 Hypothesis”, sn-2 palmitate, characterized as PA located at the sn-2 position of dietary TAGs, may raise LDL concentrations [7]. A previous study found that sn-2 palmitate had little effect on lipoprotein concentrations [8]. Specifically, there were nonsignificant increases of 0.06 mmol/L for TC, 0.03 mmol/L for HDLC, and 0.04 mmol/L for LDLC compared with the control; the increases in TC and LDLC were statistically significant in the men but not in women [8]. Conversely, Forsythe et al. [24] found that diets providing PA in the sn-2 position resulted in significantly lower TC concentrations than diets providing PA in the sn-1 and -3 positions, and no significant effect of diet was observed on HDLC or LDLC concentration. In this study, we observed a mild but significant decrease in HDLC concentration in both arms (−0.07 and −0.05 mmol/L, respectively). This finding was consistent in subgroup analysis, except for males. Menopausal status is associated with decreases in HDLC independent of aging. However, data also suggest that the cardioprotective benefits of HDLC are not only lost in postmenopausal women but may completely reverse [25]. Therefore, the results should be interpreted with caution, and future studies on HDL particle size are necessary. A slight difference in TC/HDLC ratio between groups was found (*p* = 0.049, without multiple comparison adjustment) at week 12 due to the change in HDLC, but this difference disappeared at week 24. Meanwhile, the remnant cholesterol remained stable throughout the intervention period. All in all, it can be argued that there is no impact of sn-2 palmitate on blood lipids.

In trials of infant formula feeding, it was found that formula enriched with sn-2-palmitate was able to increase body weight and length compared to normal formula [2,26], which benefits from a positive correlation between sn-2 palmitic acid content and fat absorption [27]. Malnutrition and overweight or obesity are common in middle-aged and elderly adults due to an unbalanced diet or lack of physical activity [28,29,30]. Therefore, it is important to investigate whether sn-2 palmitate intake increases body weight and fat mass. Surprisingly, from baseline to week 24, both groups experienced a weight loss of approximately 1 kg. The weight change might be due to the altered diet, which involved a reduction in the consumption of liquid milk, yogurt, nuts and seeds following the start of the intervention. However, this change remains within the acceptable normal range. Although the body fat mass remained stable, the level of visceral fat increased in females but not in males. This sex discrepancy was similar to that observed for HDLC, suggesting that sn-2 palmitate may elicit different metabolic responses in different genders, particularly in postmenopausal women. Further studies with larger sample sizes are required to elucidate its effects.

The stable body fat mass and decreased weight indicate a loss of lean mass. This was consistent with a recent meta-analysis, which found that in postmenopausal women, whey protein supplementation without resistance training had no significant benefit on muscle strength or lean mass compared to placebo controls [31]. In particular, a group × time interaction tended to be significant for SMI, and in participants without dyslipidemia, this difference was strengthened but remained insignificant. Several studies in animals and infants have confirmed that sn-2 palmitate modulates the alpha- and beta-diversity of the intestinal microbiota, promotes the abundance of gut Bifidobacteria, and increases the level of short-chain fatty acids [4,32,33,34,35]. The gut microbiota changes have been shown to directly affect muscle phenotypes; Lactobacillus and Bifidobacterium strains have restored age-related muscle loss [36]. Therefore, the intake of OPO-enriched milk powder may help to promote muscle health in middle-aged and elderly adults through the gut–muscle axis. Further and larger studies are needed to confirm this trend.

This study has some limitations. Firstly, body composition was measured by using the bioelectrical impedance analysis method instead of the standard method (dual X-ray-absorptiometry, DXA) due to limited research funding. However, this method allowed for repeated measurements over 24 weeks, which was not feasible for DXA during the current study period. Secondly, this study was an analysis of secondary outcomes, and no adjustments were performed for multiple comparisons, which increased the probability of type I error. Therefore, all tendencies or significant changes observed (e.g., time-dependent decrease in HDLC) should be judged and interpreted with caution, and further studies are required. However, as far as we know, this is the first study to investigate the impact and safety of OPO as a daily dietary option on blood metabolic indicators and body composition in middle-aged and elderly populations. The study had a relatively large sample size, good adherence, and a longer study period, which makes the results more credible.

## 5. Conclusions

In conclusion, a daily intake of sn-2-palmitate-enriched oil had no adverse effects on fasting blood glucose, lipids and body composition compared with the control oil in adults aged 45–75 years. Sn-2 palmitate may help maintain the appendicular skeletal muscle mass in individuals without dyslipidemia. These findings demonstrated the safety of sn-2 palmitate in middle-aged and older adults and may provide insight into the potential of dietary intake of OPO-enriched milk powder to reduce age-related muscle mass loss and risk of sarcopenia.

## Figures and Tables

**Table 1:** The fatty acid composition of the two fats (% total fatty acid).

Fatty Acids	OPO-Enriched Oil	Control Oil
C8:0	0	0
C10:0	0	0
C12:0	0.1	0.2
C14:0	0.6	0.8
C16:0 ^1^	28.2	28.1
C18:0	2.9	3.8
C18:1	58.8	55.0
C18:2	7.7	10.0
Others	1.7	2.1

^1^ In the OPO group, 66% in the Sn-2 position; 24% in the control group.

**Table 2:** Characteristics of the participants at baseline ^1^.

Characteristics	OPO-Enriched Oil	Control	*p* Value
Age, years	64.49 ± 6.69	64.04 ± 7.29	0.734
Gender, *n* (%)			0.869
Male	14 (25.93)	14 (24.56)	
Female	40 (74.07)	43 (75.44)	
Ethnic group, *n* (%)			0.956
Han	52 (96.30)	55 (96.49)	
Minorities	2 (3.70)	2 (3.51)	
Working status, *n* (%)			0.902
Working or housewife	9 (16.67)	10 (17.54)	
Retired	45 (83.33)	47 (82.46)	
Educational level, *n* (%)			0.662
Basic education	12 (22.22)	9 (15.79)	
Secondary education	12 (22.22)	15 (26.32)	
High education	30 (55.56)	33 (57.89)	
Monthly income per capita ^2^, *n* (%)			0.827
0~4999	11 (20.37)	11 (19.30)	
5000~9999	30 (55.56)	34 (59.65)	
≥10,000	13 (24.07)	11 (19.30)	
Smoking status, *n* (%)			0.300
Non-Smoker	49 (90.74)	48 (84.21)	
Smoker	5 (9.26)	9 (15.79)	
Second-hand smoke exposure ^2^			
<1 day per week	46 (88.46)	48 (87.27)	0.851
2–7 days per week	6 (11.54)	7 (12.73)	
Menopause in women, *n* (%)			0.449
Postmenopausal	38 (95.00)	39 (90.70)	
Not postmenopausal	2 (5.00)	4 (9.30)	
Physical activity			0.584
Lower	5 (9.26)	9 (15.79)	
Medium	38 (70.37)	37 (64.91)	
Higher	11 (20.37)	11 (19.30)	
Weight, kg	64.94 ± 9.69	66.86 ± 10.97	0.352
Body mass index, kg/m^2^	24.70 ± 2.91	25.26 ± 3.64	0.359
Systolic blood pressure, mmHg	125.90 ± 17.94	125.28 ± 18.28	0.858
Diastolic blood pressure, mmHg	79.02 ± 9.95	77.09 ± 9.08	0.291
Hypertension, *n* (%)	15 (27.78)	14 (24.56)	0.700
Diabetes, *n* (%)	2 (3.70)	7 (12.28)	0.098
Dyslipidemia, *n* (%)	15 (27.78)	19 (33.33)	0.526

^1^ Values were Mean ± SD or *n* (%). ^2^ Missing data: 1 for monthly income in control group, 2 for second-hand smoke exposure in OPO group and 2 in control group.

**Table 3:** Energy and nutrient intake of the participants at baseline (Mean ± SD).

Energy or Nutrients	OPO-Enriched Oil	Control	*p* Value
Energy and macronutrients			
Kilocalories	1731.0 ± 522.9	1664.8 ± 440.3	0.434
Protein, g	64.3 ± 26.4	64.2 ± 21.3	0.876
Fat, g	69.2 ± 25.0	70.3 ± 19.9	0.946
Carbohydrate, g	219.3 ± 66.1	198.7 ± 71.9	0.042
Micronutrients			
Fiber, g	13.1 ± 6.1	12.2 ± 6.2	0.310
Cholesterol, mg	409.1 ± 208.6	422.0 ± 170.4	0.267
Vitamin A, µgRE	619.2 ± 322.7	581.2 ± 372.0	0.224
Thiamin, mg	0.9 ± 0.9	0.8 ± 0.3	0.972
Riboflavin, mg	1.0 ± 0.4	0.9 ± 0.4	0.513
Niacin, mg	10.7 ± 6.1	10.8 ± 6.8	0.600
Vitamin C, mg	80.0 ± 36.4	76.7 ± 37.2	0.600
Vitamin E, mg	19.1 ± 35.9	16.5 ± 14.1	0.841
Ca, mg	576.4 ± 255.4	537.8 ± 385.5	0.095
P, mg	956.5 ± 402.2	904.8 ± 356.8	0.440
K, mg	1894.3 ± 737.6	1721.7 ± 663.0	0.278
Na, mg	692.6 ± 421.1	655.6 ± 421.5	0.520
Mg, mg	281.3 ± 155.3	264.9 ± 118.3	0.697
Fe, mg	15.6 ± 5.9	16.2 ± 7.7	0.948
Zn, mg	8.4 ± 3.7	8.2 ± 3.8	0.732
Se, µg	40.6 ± 18.5	39.9 ± 16.1	0.944
Cu, mg	1.5 ± 0.7	1.5 ± 1.0	0.339
Mn, mg	3.9 ± 1.6	3.6 ± 1.8	0.262

**Table 4:** Analysis for control compared with OPO group; estimated marginal means (SE) for metabolic biomarkers.

Indicators	OPO Group			Control Group			*p* Value		
	Baseline	Week 12	Week 24	Baseline	Week 12	Week 24	Group	Time	Group × Time
Glucose, mmol/L	5.42 ± 0.22	5.48 ± 0.22	5.50 ± 0.22	5.41 ± 0.15	5.47 ± 0.15	5.56 ± 0.15	0.951	0.149	0.791
Triglyceride, mmol/L	1.28 ± 0.094	1.32 ± 0.095	1.32 ± 0.097	1.30 ± 0.091	1.28 ± 0.091	1.29 ± 0.093	0.888	0.928	0.842
TyG index	8.48 ± 0.077	8.56 ± 0.077	8.57 ± 0.079	8.52 ± 0.070	8.54 ± 0.071	8.56 ± 0.071	0.976	0.196	0.665
Total Cholesterol, mmol/L	4.97 ± 0.15	5.00 ± 0.15	5.00 ± 0.15	4.97 ± 0.14	4.78 ± 0.14	4.86 ± 0.14	0.480	0.518	0.256
HDLC, mmol/L	1.44 ± 0.054	1.39 ± 0.054	1.37 ± 0.054	1.41 ± 0.046	1.39 ± 0.047	1.36 ± 0.047	0.832	0.003	0.575
LDLC, mmol/L	2.95 ± 0.13	3.02 ± 0.13	3.03 ± 0.13	2.97 ± 0.13	2.81 ± 0.13	2.91 ± 0.13	0.504	0.664	0.184
Remnant Cholesterol, mmol/L	0.58 ± 0.043	0.60 ± 0.043	0.60 ± 0.044	0.59 ± 0.041	0.58 ± 0.042	0.59 ± 0.042	0.885	0.936	0.843
TC/HDLC ratio	3.61 ± 0.14	3.77 ± 0.14	3.82 ± 0.15	3.69 ± 0.15	3.58 ± 0.15	3.69 ± 0.15	0.646	0.172	0.071

**Table 5:** Analysis for control compared with OPO group; estimated marginal means (SE) for body composition indicators.

Indicators	OPO Group				Control Group				*p* Value		
	Baseline	Week 4	Week 12	Week 24	Baseline	Week 4	Week 12	Week 24	Group	Time	Group × Time
Weight, kg	67.80 ± 1.14	67.98 ± 1.14	67.28 ± 1.14	66.77 ± 1.14	69.92 ± 1.36	70.14 ± 1.36	69.33 ± 1.36	68.96 ± 1.36	0.195	<0.001	0.968
Body mass index, kg/m^2^	24.88 ± 0.41	24.95 ± 0.41	24.70 ± 0.41	24.51 ± 0.41	25.53 ± 0.50	25.61 ± 0.50	25.29 ± 0.50	25.14 ± 0.50	0.292	<0.001	0.936
Total body water, kg	33.91 ± 0.54	33.90 ± 0.54	33.79 ± 0.54	32.93 ± 0.55	34.03 ± 0.49	33.91 ± 0.49	33.87 ± 0.49	33.34 ± 0.50	0.806	0.003	0.862
Protein, kg	9.00 ± 0.14	8.98 ± 0.14	8.96 ± 0.14	8.72 ± 0.14	9.02 ± 0.13	9.00 ± 0.13	8.98 ± 0.13	8.83 ± 0.13	0.795	0.002	0.871
Soft lean mass, kg	43.47 ± 0.70	43.44 ± 0.70	43.29 ± 0.70	42.19 ± 0.70	43.59 ± 0.62	43.47 ± 0.63	43.40 ± 0.63	42.71 ± 0.63	0.806	0.002	0.870
Fat-free mass, kg	46.09 ± 0.73	46.04 ± 0.73	45.88 ± 0.73	44.73 ± 0.74	46.16 ± 0.65	46.10 ± 0.66	46.01 ± 0.66	45.30 ± 0.66	0.804	0.002	0.850
Skeletal muscle, kg	25.19 ± 0.43	25.11 ± 0.43	25.03 ± 0.43	24.32 ± 0.44	25.19 ± 0.38	25.17 ± 0.39	25.11 ± 0.39	24.62 ± 0.39	0.820	<0.001	0.883
Skeletal muscle index, kg/m^2^	7.13 ± 0.10	7.11 ± 0.09	7.11 ± 0.09	7.02 ± 0.10	7.40 ± 0.13	7.16 ± 0.13	7.05 ± 0.13	6.95 ± 0.14	0.697	0.003	0.090
Body fat mass, kg	21.58 ± 0.85	21.81 ± 0.85	21.24 ± 0.85	21.93 ± 0.86	23.62 ± 0.96	23.88 ± 0.97	23.18 ± 0.97	23.50 ± 0.97	0.094	0.286	0.886
Percent body fat, %	31.93 ± 0.92	32.33 ± 0.92	31.77 ± 0.92	33.20 ± 0.93	33.66 ± 0.84	33.83 ± 0.85	33.27 ± 0.85	33.94 ± 0.86	0.183	0.177	0.763
InBody score	67.76 ± 1.00	67.54 ± 1.00	67.95 ± 1.00	66.03 ± 1.02	65.72 ± 0.89	65.39 ± 0.91	66.09 ± 0.90	65.11 ± 0.93	0.082	0.175	0.806
Visceral fat level	8.38 ± 0.34	8.97 ± 0.34	8.72 ± 0.34	8.94 ± 0.35	9.13 ± 0.37	9.31 ± 0.37	9.49 ± 0.37	9.72 ± 0.37	0.118	0.017	0.564

## Data Availability

The data presented in this study are available on request from the corresponding author. The data are not publicly available due to ethical requirements.

## Supplementary Materials

The following supporting information can be downloaded at: https://www.mdpi.com/article/10.3390/nu16070973/s1.
