# Supplementary material for: The Effect of Sn-2 Palmitate on Blood Glucose, Lipids and Body Composition in Middle-Aged and Elderly Adults: A Randomized, Double-Blinded Controlled Trial
Source: Nutrients. 2024 Mar 27;16(7):973. doi: 10.3390/nu16070973 (PMC11013204; doi:10.3390/nu16070973)
Supplement: Supplementary file 1 [file nutrients-16-00973-s001.zip › nutrients-2891470-supplementary.pdf]

nutrients MDPI

Supplemental materials

The Effect of Sn-2 Palmitate on Blood Glucose, Lipids and Body Composition in Middle-Aged and Elderly Adults: A Randomized, Double-Blinded Controlled Trial

Figure S1. The trajectories of adjusted mean changes in body composition indicators during the study in two arms 2

Tabel S1. The main food intake in both groups at baseline (Median, Interquartile Range). 3

Tabel S2. Effect of OPO on blood glucose and lipids. 3

Table S3. Effect of OPO on body composition. 3

Table S4. Subgroup Analysis[a] for OPO compared with control on blood glucose, lipids and body composition indicators in male and female. 4

Table S5. Effect of OPO on HDLC and SMI in subgroups. 5

Table S6. Subgroup Analysis[a] for OPO compared with control on blood glucose, lipids and body composition indicators in participants with/without dyslipidemia. 5

Table S7. Estimated marginal means for physical activity levels and food intake in OPO and control groups... 6

Nutrients **2024**, *16*, 973. https://doi.org/10.3390/nu16070973 www.mdpi.com/journal/nutrients

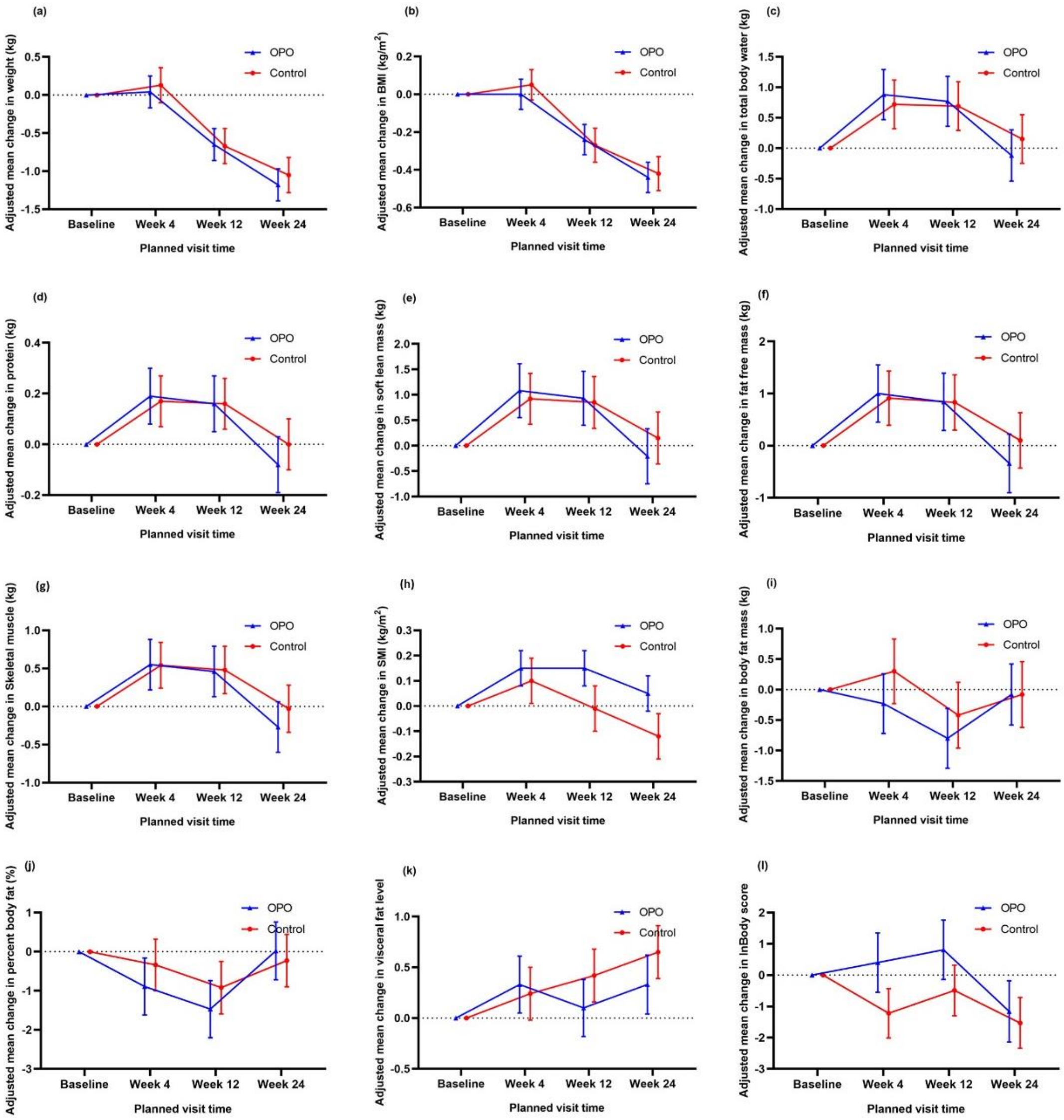

Figure S1. The trajectories of adjusted mean changes in body composition indicators during the study in two arms. (**a**) body weight; (**b**) BMI; (**c**) total body water; (**d**) protein; (**e**) soft lean mass; (**f**) fat free mass; (**g**) skeletal muscle; (**h**) SMI; (**i**) body fat mass; (**j**) percent body fat; (**k**) visceral fat level; (**l**) InBody score; BMI, body mass index; SMI, skeletal mass index.

Table S1. The main food intake in both groups at baseline (Median, Interquartile Range).

Food groups	OPO	Control	*p* Value
Staple foods	226.8 (154.2–339.6)	217.2 (147.1–310.0)	0.280
Vegetables	219.6 (133.7–436.8)	275.1 (168.8–451.8)	0.584
Fruits	189.3 (100.0–300.0)	150.0 (120.0–300.0)	0.745
Mushrooms	7.4 (2.7–17.8)	6.7 (1.3–12.9)	0.345
Nuts and seeds	15.0 (3.0–30.0)	10.7 (1.4–25.0)	0.423
Soybean	14.3 (5.4–28.6)	13.4 (1.9–21.4)	0.374
Livestock and poultry meat	46.8 (25.2–100.0)	50.0 (25.0–100.0)	0.719
Sea foods	14.5 (7.0–31.9)	18.3 (6.9–38.7)	0.757
Eggs	50.0 (50.0–63.8)	60.0 (50.0–75.0)	0.133
Liquid milk	178.6 (45.5–250.0)	107.1 (0.0–240.0)	0.191
Yogurt	60.7 (13.6–150.0)	19.3 (0.0–71.4)	**0.010**
Milk powder	0.0 (0.0–0.0)	0.0 (0.0–0.0)	0.498

Table S2. Effect of OPO on blood glucose and lipids.

Endpoints, Change from Baseline	Time	Adjusted [a] Mean (SE)		Adjusted [a] Mean Difference (95% Confidence Interval)	*p* Value
		OPO	Control		
Glucose, mmol/L	Week 12	0.04 (0.07)	0.05 (0.10)	−0.01 (−0.25, 0.23)	0.936
	Week 24	0.06 (0.08)	0.15 (0.11)	−0.09 (−0.34, 0.15)	0.462
Triglyceride, mmol/L	Week 12	0.05 (0.06)	0.03 (0.07)	0.03 (−0.14, 0.20)	0.746
	Week 24	0.04 (0.06)	0.04 (0.07)	0.00 (−0.18, 0.18)	0.963
TyG	Week 12	0.10 (0.05)	0.05 (0.05)	0.04 (−0.09, 0.18)	0.512
	Week 24	0.10 (0.05)	0.08 (0.06)	0.01 (−0.13, 0.15)	0.841
Total Cholesterol, mmol/L	Week 12	0.06 (0.09)	−0.15 (0.10)	0.21 (−0.04, 0.46)	0.104
	Week 24	0.07 (0.10)	−0.06 (0.10)	0.13 (−0.12, 0.39)	0.306
HDLC, mmol/L	Week 12	−0.06 (0.02)	−0.03 (0.03)	−0.03 (−0.09, 0.03)	0.389
	Week 24	−0.07 (0.02)	−0.05 (0.03)	−0.02 (−0.09, 0.04)	0.466
LDLC, mmol/L	Week 12	0.10 (0.09)	−0.12 (0.09)	0.22 (−0.01, 0.45)	0.066
	Week 24	0.12 (0.09)	−0.02 (0.09)	0.14 (−0.10, 0.38)	0.240
Remnant Cholesterol, mmol/L	Week 12	0.02 (0.03)	0.01 (0.03)	0.01 (−0.07, 0.09)	0.749
	Week 24	0.02 (0.03)	0.02 (0.03)	0.00 (−0.08, 0.08)	0.961
TC/HDLC ratio	Week 12	0.21 (0.08)	−0.02 (0.09)	0.22 (0.00, 0.45)	**0.049**
	Week 24	0.26 (0.08)	0.08 (0.09)	0.18 (−0.04, 0.41)	0.114

[a] Adjusted for age and gender. TyG, triglyceride-glucose; HDLC, high-density lipoprotein cholesterol; LDLC, low-density lipoprotein cholesterol. TC, total cholesterol.

Table S3. Effect of OPO on body composition.

Endpoints, Change from Baseline	Time	Adjusted [a] Mean (SE)		Adjusted [a] Mean Difference (95% Confidence Interval)	*p* Value
		OPO	Control		
Weight, kg	Week 4	0.04 (0.21)	0.13 (0.23)	−0.09 (−0.65, 0.47)	0.751
	Week 12	−0.65 (0.21)	−0.67 (0.23)	0.02 (−0.54, 0.59)	0.932
	Week 24	−1.18 (0.21)	−1.05 (0.23)	−0.13 (−0.71, 0.44)	0.655
Body mass index, kg/m^2	Week 4	0.00 (0.08)	0.05 (0.08)	−0.04 (−0.25, 0.17)	0.698
	Week 12	−0.24 (0.08)	−0.27 (0.09)	0.03 (−0.18, 0.24)	0.784
	Week 24	−0.44 (0.08)	−0.42 (0.09)	−0.02 (−0.23, 0.20)	0.890
Total body water, kg	Week 4	0.88 (0.41)	0.72 (0.40)	0.16 (−0.82, 1.14)	0.745
	Week 12	0.77 (0.41)	0.69 (0.40)	0.08 (−0.90, 1.06)	0.873
	Week 24	−0.12 (0.42)	0.15 (0.40)	−0.27 (−1.27, 0.73)	0.598
Protein, kg	Week 4	0.19 (0.11)	0.17 (0.10)	0.02 (−0.24, 0.27)	0.894
	Week 12	0.16 (0.11)	0.16 (0.10)	0.01 (−0.25, 0.26)	0.956
	Week 24	−0.08 (0.11)	0.00 (0.10)	−0.08 (−0.34, 0.18)	0.540
Soft lean mass, kg	Week 4	1.08 (0.53)	0.92 (0.50)	0.16 (−1.09, 1.41)	0.798
	Week 12	0.93 (0.53)	0.85 (0.51)	0.08 (−1.17, 1.33)	0.904
	Week 24	−0.21 (0.54)	0.15 (0.51)	−0.36 (−1.63, 0.92)	0.580

Endpoints, Change from Baseline	Time	Adjusted [a] Mean (SE)		Adjusted [a] Mean Difference (95% Confidence Interval)	*p* Value
		OPO	Control		
Fat free mass, kg	Week 4	1.00 (0.55)	0.91 (0.52)	0.09 (−1.20, 1.39)	0.889
	Week 12	0.84 (0.55)	0.83 (0.53)	0.01 (−1.29, 1.30)	0.990
	Week 24	−0.34 (0.56)	0.10 (0.53)	−0.44 (−1.76, 0.88)	0.509
Skeletal muscle, kg	Week 4	0.55 (0.33)	0.54 (0.30)	0.01 (−0.75, 0.77)	0.981
	Week 12	0.46 (0.33)	0.48 (0.31)	−0.02 (−0.78, 0.74)	0.957
	Week 24	−0.27 (0.33)	−0.03 (0.31)	−0.24 (−1.02, 0.53)	0.537
Skeletal muscle index, kg/m^2	Week 4	0.15 (0.07)	0.10 (0.09)	0.05 (−0.15, 0.25)	0.626
	Week 12	0.15 (0.07)	−0.01 (0.09)	0.16 (−0.04, 0.36)	0.124
	Week 24	0.05 (0.07)	−0.12 (0.09)	0.17 (−0.04, 0.37)	0.112
Body fat mass, kg	Week 4	−0.23 (0.49)	0.30 (0.53)	−0.53 (−1.88, 0.82)	0.441
	Week 12	−0.80 (0.49)	−0.42 (0.54)	−0.38 (−1.73, 0.97)	0.577
	Week 24	−0.08 (0.50)	−0.08 (0.54)	0.00 (−1.37, 1.38)	0.994
Percent body fat, %	Week 4	−0.89 (0.73)	−0.34 (0.66)	−0.54 (−2.35, 1.26)	0.553
	Week 12	−1.47 (0.73)	−0.92 (0.67)	−0.54 (−2.35, 1.26)	0.554
	Week 24	0.02 (0.74)	−0.23 (0.67)	0.25 (−1.60, 2.10)	0.789
InBody score	Week 4	0.40 (0.95)	−1.22 (0.79)	1.62 (−0.71, 3.94)	0.172
	Week 12	0.81 (0.95)	−0.49 (0.81)	1.30 (−1.03, 3.62)	0.273
	Week 24	−1.16 (0.98)	−1.53 (0.81)	0.37 (−2.01, 2.75)	0.760
Visceral fat level	Week 4	0.33 (0.28)	0.24 (0.26)	0.09 (−0.62, 0.80)	0.808
	Week 12	0.10 (0.28)	0.42 (0.26)	−0.32 (−1.03, 0.39)	0.378
	Week 24	0.33 (0.29)	0.65 (0.26)	−0.32 (−1.04, 0.41)	0.388

[a] Adjusted for age and gender.

Table S4. Subgroup Analysis[a] for OPO compared with control on blood glucose, lipids and body composition indicators in male and female.

Endpoints	*p* Value for Male (n = 28)			*p* Value for Female (n = 83)		
	Group	Time	Group * Time	Group	Time	Group * Time
**Blood glucose and lipids**						
Glucose, mmol/L	0.686	0.715	0.564	0.772	0.123	0.941
Triglyceride, mmol/L	0.047	0.725	0.212	0.328	0.996	0.833
TyG	0.064	0.152	0.145	0.553	0.559	0.556
Total Cholesterol, mmol/L	0.902	0.228	0.346	0.507	0.109	0.538
HDLC, mmol/L	0.255	0.911	0.202	0.846	0.000	0.579
LDLC, mmol/L	0.776	0.233	0.303	0.606	0.362	0.420
Remnant Cholesterol, mmol/L	0.047	0.749	0.210	0.325	0.995	0.829
TC/HDLC	0.226	0.325	0.077	0.322	0.322	0.265
**Body composition indicators**						
Weight, kg	0.565	<0.001	0.631	0.246	<0.001	0.730
Body mass index, kg/m^2	0.435	<0.001	0.593	0.408	<0.001	0.547
Total body water, kg	0.500	0.001	0.630	0.380	0.089	0.771
Protein, kg	0.507	0.001	0.529	0.377	0.060	0.803
Soft lean mass, kg	0.504	0.001	0.627	0.385	0.083	0.797
Fat free mass, kg	0.505	0.001	0.619	0.381	0.089	0.807
Skeletal muscle, kg	0.507	0.000	0.532	0.402	0.045	0.856
Skeletal muscle index, kg/m^2	0.407	0.013	0.548	0.436	0.007	0.107
Body fat mass, kg	0.083	0.766	0.637	0.288	0.330	0.672
Percent body fat, %	0.065	0.474	0.670	0.546	0.245	0.599
InBody score	0.036	0.198	0.696	0.407	0.293	0.682
Visceral fat level	0.102	0.477	0.792	0.389	0.007	0.630

[a] Adjusted for age and gender.

Table S5. Effect of OPO on HDLC and SMI in subgroups.

Endpoints, Change from Baseline	Subgroups	Time	Adjusted [a] Mean (SE)		Adjusted [a] Mean Difference (95% Confidence Interval)	*p* Value
			OPO	Control		
HDLC, mmol/L	Male	Week 12	0.00 (0.04)	−0.02 (0.06)	0.02 (−0.14, 0.17)	0.796
		Week 24	−0.03 (0.04)	0.05 (0.07)	−0.08 (−0.25, 0.09)	0.335
	Female	Week 12	−0.07 (0.02)	−0.04 (0.02)	−0.03 (−0.10, 0.03)	0.299
		Week 24	−0.08 (0.02)	−0.08 (0.02)	0.00 (−0.07, 0.07)	0.982
	Dyslipidemia	Week 12	−0.02 (0.03)	0.01 (0.04)	−0.03 (−0.12, 0.07)	0.587
		Week 24	−0.06 (0.04)	−0.12 (0.04)	0.05 (−0.06, 0.17)	0.356
	Non-dyslipidemia	Week 12	−0.07 (0.02)	−0.04 (0.03)	−0.03 (−0.10, 0.05)	0.490
		Week 24	−0.07 (0.02)	−0.02 (0.03)	−0.05 (−0.13, 0.02)	0.174
SMI, kg/m^2	Male	Week 4	0.13 (0.06)	−0.02 (0.07)	0.15 (−0.04, 0.34)	0.109
		Week 12	0.04 (0.06)	−0.07 (0.08)	0.11 (−0.08, 0.30)	0.240
		Week 24	−0.10 (0.06)	−0.15 (0.08)	0.05 (−0.15, 0.24)	0.617
	Female	Week 4	−0.22 (0.07)	−0.20 (0.10)	−0.02 (−0.27, 0.23)	0.863
		Week 12	−0.19 (0.07)	−0.33 (0.10)	0.14 (−0.11, 0.38)	0.277
		Week 24	−0.27 (0.08)	−0.44 (0.10)	0.17 (−0.08, 0.42)	0.188
	Dyslipidemia	Week 4	−0.02 (0.07)	0.05 (0.09)	−0.07 (−0.25, 0.12)	0.454
		Week 12	−0.05 (0.06)	−0.05 (0.09)	0.00 (−0.18, 0.19)	0.966
		Week 24	−0.13 (0.07)	−0.18 (0.09)	0.05 (−0.14, 0.24)	0.601
	Non-dyslipidemia	Week 4	0.11 (0.09)	−0.01 (0.10)	0.12 (−0.13, 0.37)	0.352
		Week 12	0.12 (0.09)	−0.12 (0.10)	0.24 (−0.01, 0.49)	0.059
		Week 24	0.01 (0.09)	−0.22 (0.10)	0.23 (−0.02, 0.48)	0.067

[a] Adjusted for age and gender.

Table S6. Subgroup Analysis[a] for OPO compared with control on blood glucose, lipids and body composition indicators in participants with/without dyslipidemia.

Endpoints	*p* Value for Participants with Dyslipidemia (n = 34)			*p* Value for Participants without Dyslipidemia (n = 77)		
	Group	Time	Group * Time	Group	Time	Group * Time
**Blood glucose and lipids**						
Glucose, mmol/L	0.662	0.092	0.210	0.666	0.378	0.356
Triglyceride, mmol/L	0.773	0.616	0.712	0.904	0.572	0.983
TyG	0.974	0.789	0.182	0.977	0.078	0.988
Total Cholesterol, mmol/L	0.604	0.417	0.613	0.397	0.890	0.171
HDLC, mmol/L	0.480	0.008	0.537	0.700	0.012	0.532
LDLC, mmol/L	0.266	0.606	0.448	0.242	0.984	0.074
Remnant Cholesterol, mmol/L	0.770	0.616	0.708	0.906	0.594	0.983
TC/HDLC	0.517	0.258	0.370	0.308	0.253	0.018
**Body composition indicators**						
Weight, kg	0.113	0.012	0.378	0.877	<0.001	0.898
Body mass index, kg/m^2	0.164	0.014	0.342	0.827	<0.001	0.984
Total body water, kg	0.395	0.001	0.219	0.579	0.062	0.502
Protein, kg	0.377	0.001	0.349	0.615	0.044	0.574
Soft lean mass, kg	0.391	0.001	0.227	0.582	0.055	0.530
Fat free mass, kg	0.370	0.001	0.215	0.569	0.059	0.530
Skeletal muscle, kg	0.373	0.000	0.250	0.582	0.028	0.621
Skeletal muscle index, kg/m^2	0.288	0.001	0.589	0.777	0.011	0.111
Body fat mass, kg	0.060	0.224	0.327	0.800	0.171	0.722
Percent body fat, %	0.165	0.016	0.272	0.686	0.257	0.602
Inbody score	0.060	0.023	0.241	0.388	0.305	0.570
Visceral fat level	0.112	0.009	0.050	0.886	0.030	0.857

[a] Adjusted for age and gender.

Table S7. Estimated marginal means for physical activity levels and food intake in OPO and control groups.

Endpoints	Time	Adjusted [a] Mean (SE) or Mean (95%CI)		*p* Value		
		OPO	Control	Group	Time	Group * Time
Physical activity[b]	Baseline	7.36 (0.20)	7.33 (0.20)	0.298	0.208	0.473
	Week 4	7.12 (0.20)	7.07 (0.20)			
	Week 12	7.29 (0.20)	6.78 (0.20)			
	Week 24	7.09 (0.21)	6.95 (0.20)			
Staple foods	Baseline	303.28 (19.07)	274.49 (16.36)	0.199	0.578	0.876
	Week 4	288.64 (19.34)	282.67 (16.46)			
	Week 12	284.45 (19.35)	258.29 (16.57)			
	Week 24	284.41 (19.78)	262.47 (16.91)			
Vegetables[c]	Baseline	247.7 (202.1 - 363.4)	256.8 (208.0 - 317.6)	0.469	0.080	0.247
	Week 4	196.2 (159.7 - 241.9)	250.7 (202.8 - 339.7)			
	Week 12	219.5 (178.7 - 269.5)	242.5 (195.9 - 399.3)			
	Week 24	213.5 (173.2 - 263.1)	198.1 (159.5 - 245.9)			
Fruits[c]	Baseline	148.5 (122.2–180.5)	146.2 (111.0–192.4)	0.583	0.056	0.435
	Week 4	128.6 (105.5–156.6)	137.9 (104.3–182.3)			
	Week 12	154.4 (126.7–188.2)	122.4 (92.5–162.1)			
	Week 24	178.1 (145.6–217.9)	162.8 (122.4–216.4)			
Nuts and seeds[c]	Baseline	12.7 (8.5–18.9)	9.9 (6.6–14.9)	0.351	<0.001	0.514
	Week 4	10.5 (7.0–15.7)	7.2 (4.8–10.9)			
	Week 12	7.7 (5.2–11.6)	6.0 (4.0–9.1)			
	Week 24	6.1 (4.0–9.1)	5.4 (3.5–8.2)			
Liquid milk[c]	Baseline	54.3 (31.3–94.2)	32.4 (19.0–55.3)	0.430	<0.001	0.585
	Week 4	3.1 (1.8–5.4)	3.1 (1.8–5.3)			
	Week 12	2.9 (1.7–5.0)	2.1 (1.2–3.6)			
	Week 24	1.8 (1.0–3.1)	1.7 (1.0–3.0)			
Yogurt[c]	Baseline	29.3 (16.1–53.2)	10.5 (5.9–18.6)	0.005	<0.001	0.701
	Week 4	14.1 (7.7–25.8)	5.0 (2.8–8.9)			
	Week 12	14.8 (8.1–27.1)	5.4 (3.0–9.6)			
	Week 24	10.4 (5.6–19.2)	5.6 (3.1–10.1)			
Milk powder[c]	Baseline	1.6 (1.3–1.9)	2.0 (1.6–2.4)	0.239	<0.001	0.452
	Week 4	36.6 (30.5–44.0)	36.8 (29.8–45.4)			
	Week 12	34.1 (28.4–40.9)	39.7 (32.1–49.0)			
	Week 24	33.9 (28.1–40.8)	32.3 (26.1–40.1)			

[a] Adjusted for age and gender. [b] Log-transformed value. [c] Values were log-transformed, and the results (mean, 95%CI) were re-transformed.
